# Comparative Outcomes of Open, Laparoscopic, and Robotic Right Colectomy for Colon Cancer: Postoperative Recovery, Surgical Quality, and 3-Year Survival—A Monocentric Retrospective Study

**DOI:** 10.3390/cancers18172769

**Published:** 2026-08-26

**Authors:** Krzysztof Nowakowski, Leonie Wesselmann, Michael Leitz, Fabian Nimczewski, Annika Hoyer, Kira Baginski, Wolfgang Hiller, Jens Hoeppner, Zsolt Madarasz

**Affiliations:** 1 Department of Surgery, Medical School and University Medical Center OWL, Bielefeld University, Campus Lippe, Röntgenstr. 18, 32756 Detmold, Germany; 2 Biostatistics and Medical Biometry, Medical School OWL, Bielefeld University, 33615 Bielefeld, Germany

**Keywords:** colon cancer, right colectomy, robotic surgery, minimally invasive surgery, laparoscopy, complete mesocolic excision, postoperative recovery, survival

## Abstract

Background/Objectives: Minimally invasive approaches are increasingly used in right colectomy for colon cancer; however, comparative data, including open, laparoscopic, and robotic techniques, remain limited, particularly regarding postoperative recovery and oncological outcomes. Methods: This retrospective monocentric study included 89 patients undergoing elective right colectomy for colon cancer between July 2018 and December 2022. Of these, 30 patients underwent open, 29 laparoscopic, and 30 robotic-assisted surgery. The primary endpoint was postoperative recovery, defined as the time to first bowel movement. Secondary endpoints included length of hospital stay, complete mesocolic excision (CME) quality, lymph node yield, C-reactive protein (CRP) on postoperative day one, operative time, overall survival (OS), and disease-free survival (DFS). Multivariable regression analyses were performed adjusting for age, sex, BMI, ASA score, UICC stage, and tumor size. Results: Time to first bowel movement was shorter after minimally invasive surgery (median robotic 3.5, laparoscopic 4 vs. open 5 days) and length of hospital stay was numerically shorter (mean 9.1 vs. 10.1 days). CME quality and lymph node yield did not differ significantly between the approaches, and postoperative day-one CRP was numerically lower after robotic (median 4.9 mg/dL) and laparoscopic (5.1 mg/dL) than after open surgery (7.1 mg/dL), without reaching statistical significance after adjustment. Operative time was significantly longer with minimally invasive surgery (robotic +50 min; laparoscopic +43 min vs. open; both *p* < 0.001). Disease-free (open 51.6%, laparoscopic 66.5%, robotic 66.9%) and overall survival did not differ significantly after adjustment (robotic HR 1.16; laparoscopic HR 1.01 vs. open). Conclusions: No statistically significant difference in surgical quality between the approaches was detected, while operative time was significantly longer after minimally invasive surgery. No statistically significant independent association between the surgical approach and postoperative recovery or three-year oncological outcomes was detected; given the limited precision of the estimates, clinically important differences cannot be excluded.

## Simple Summary

Surgery is the main treatment for cancer of the right side of the colon, and it can be performed in three ways: through a large open incision, with keyhole (laparoscopic) surgery, or with the assistance of a surgical robot. It remains unclear whether the newer minimally invasive techniques offer real advantages over open surgery in terms of recovery, the quality of the cancer operation, and long-term survival. In this study, we reviewed 89 patients treated at a single German university hospital and compared the three approaches. Patients recovered somewhat faster after keyhole and robotic surgery, and no difference in the quality of the cancer removal between the three methods could be demonstrated, although the minimally invasive operations took longer. Once differences between patients were taken into account, we could not demonstrate an independent effect of the surgical approach on recovery or three-year survival, although the study was too small to rule out meaningful differences. These findings suggest that all three techniques are reasonable options and that the choice should be guided by the individual patient and the surgeon’s experience.

## 1. Introduction

Right-sided colon cancer represents approximately 33% of colorectal malignancies [1] and poses specific surgical challenges due to vascular anatomy and embryological planes [2]. For decades, open right colectomy was considered the gold standard surgical therapy for right-sided colon cancer [3]. With the progress of laparoscopy [4] and, more recently, robotic-assisted surgery [5], minimally invasive techniques have increasingly been adopted in colorectal surgical oncology.

Laparoscopic right colectomy has demonstrated advantages in postoperative recovery, including reduced pain, earlier bowel function, and shorter hospital stay [3,6]. Robotic-assisted surgery was introduced to overcome limitations of laparoscopy, offering three-dimensional visualization, articulated instruments, and improved ergonomics. Nevertheless, concerns arose regarding longer operative times, higher costs, and limited long-term oncological data [5].

Comparative studies often evaluate only two techniques, and few investigations have directly compared open, laparoscopic, and robotic right colectomy within a single institution [6,7]. Survival outcomes are often underreported or limited by short follow-up. The present study compares perioperative and oncological outcomes of all three surgical approaches in a monocentric cohort.

## 2. Materials and Methods

### 2.1. Study Design and Patients

This retrospective observational study included patients undergoing elective right colectomy for histologically confirmed colon cancer between July 2018 and December 2022 at the Department of Surgery, Medical School OWL, Bielefeld University, Campus Klinikum Lippe, Detmold. Emergency procedures and extended right colectomies were excluded. All patients fulfilling these criteria during the study period were included consecutively; no patient was excluded for reasons related to the operative approach. The choice of approach was not randomised and was made by the operating surgeon on the basis of tumour characteristics, previous abdominal surgery, anticipated technical difficulty, comorbidity, and availability of the robotic platform, which was introduced at our institution in February 2021; the resulting distribution of procedures by calendar year and by surgeon is reported in Supplementary Tables S14 and S15. Postoperative surveillance followed the institutional protocol for colon cancer, implementing the programme of the German S3 guideline for colorectal cancer [8]: history and clinical examination at 6, 12, 18, 24, 36, 48, and 60 months after surgery; abdominal ultrasound at 12, 24, 36, 48, and 60 months, with spiral computed tomography as an alternative imaging modality at 12 months where individually indicated; and complete colonoscopy at 12 months and again at 60 months if unremarkable, with an additional colonoscopy at 6 months in patients in whom a complete colonoscopy had not been performed preoperatively. Recurrence was defined as radiologically or histologically confirmed local, locoregional, or distant tumour recurrence. Follow-up data were obtained from institutional clinical records and from the regional clinical cancer registry. Five patients (5.6%) did not complete 36 months of observation and were censored at the date of last documented contact.

### 2.2. Preoperative Management

All patients received standardized preoperative bowel preparation with orthograde lavage using CitraFleet^®^ (sodium picosulfate/magnesium citrate; Casen Recordati, S.L., Utebo, Zaragoza, Spain) (two sachets dissolved in 150 mL water each), followed by ingestion of 1.5–2.0 L of clear fluids after each sachet. Twelve hours before surgery, oral antibiotic prophylaxis with paromomycin (Humatin^®^, paromomycin sulfate, Pfizer Pharma GmbH, Berlin, Germany, 8 g) was administered. Intravenous antibiotic prophylaxis with ampicillin–sulbactam 3 g (Unacid^®^, Pfizer Pharma GmbH, Berlin, Germany) was given 30 min before skin incision.

### 2.3. Operative Techniques

#### 2.3.1. Open Right Colectomy

Open right colectomy was performed via midline laparotomy with the patient in supine position. After team time-out, exploratory laparotomy was conducted to exclude peritoneal carcinomatosis and liver metastases. Dissection followed a medial-to-lateral approach along embryological planes with complete mesocolic excision (CME) and central vascular ligation [9]. The ileocolic vein and artery were identified at their origins and divided using a high-tie technique. Dissection was continued cranially along the superior mesenteric vein, with ligation of branches of the Henle trunk as required. The right branch of the middle colic artery was ligated. The lesser sac was entered, the greater omentum divided, and the right hemicolon fully mobilized, including dorsal mobilization from the Gerota fascia. The terminal ileum and transverse colon were transected using linear staplers. Bowel perfusion was assessed using indocyanine green (ICG) fluorescence angiography. Reconstruction was performed via isoperistaltic side-to-side ileotransversostomy. The mesenteric defect and abdominal fascia were closed, followed by intracutaneous skin closure.

#### 2.3.2. Laparoscopic Right Colectomy

Patients were positioned supine with split legs. Pneumoperitoneum (12 mmHg) was established via a left-sided mini-laparotomy and trocar placement. After exploratory laparoscopy, a standardized medial approach following the open-book principle [9] was used. The ileocolic vein and artery were clipped and divided centrally. Dissection proceeded in the avascular plane between the mesocolon and Gerota fascia, with blunt mobilization of the duodenum. The right branch of the middle colic artery and, if present, the right colic artery were divided. The lesser sac was opened, the transverse colon transected, and the right hemicolon fully mobilized. The terminal ileum was divided approximately 10 cm proximal to the ileocecal valve. Bowel perfusion was assessed using ICG fluorescence angiography. An intracorporeal isoperistaltic side-to-side stapled ileotransversostomy was constructed, and the mesenteric defect closed. Specimen extraction was performed via a Pfannenstiel incision, with fascial and skin closure.

#### 2.3.3. Robotic-Assisted Right Colectomy

Robotic procedures were performed using the da Vinci X^®^ Surgical System (Intuitive Surgical, Inc., Sunnyvale, CA, USA) with standardized trocar placement. Pneumoperitoneum was established using a Veres needle (Aesculap AG, Tuttlingen, Germany), followed by docking of the robotic system. Patients were positioned in moderate Trendelenburg with left-sided tilt. Dissection followed a medial approach with CME and central vascular ligation [9]. The ileocolic vein and artery were clipped and divided centrally. In the laparoscopic procedure, the right branch of the middle colic artery and, if present, the right colic artery were divided. The lesser sac was entered, the transverse colon transected with a stapler, and dissection continued along embryological planes between the mesocolon and Gerota fascia. The terminal ileum was divided using a stapler. ICG fluorescence angiography (Firefly^®^ mode, Fluorescence Imaging, Intuitive Surgical, Inc., Sunnyvale, CA, USA) was used to assess bowel perfusion. An intracorporeal isoperistaltic side-to-side ileotransversostomy was constructed using a hand-sewing technique, and the mesenteric defect was closed. Specimen extraction was performed via a Pfannenstiel incision, followed by layered closure.

### 2.4. Postoperative Management

On the day of surgery, patients were allowed oral fluids and early mobilization. The urinary catheter was removed no later than postoperative day one. From postoperative day one onward, patients received liquid nutrition, advancing to a light diet after the first bowel movement. Laboratory testing was routinely performed on postoperative days one and four. Daily physiotherapy was provided until discharge.

### 2.5. Primary Endpoint

The primary endpoint was postoperative recovery, assessed by the first postoperative bowel movement, defined as the number of days from the day of surgery to the first documented bowel movement.

### 2.6. Secondary Endpoints

Secondary endpoints included length of hospital stay, lymph nodes retrieved from the resected specimen, C-reactive protein (CRP) on postoperative day one, operative time, the quality of complete mesocolic excision (CME) according to the Benz–Tannapfel classification [10], overall survival (OS), and disease-free survival (DFS). Length of hospital stay was defined as the number of days from the day of surgery to the day of actual discharge, rather than to the day on which discharge criteria were first met; discharge required tolerance of solid food, passage of stool, pain controlled on oral analgesia, and the absence of clinical or laboratory signs of infection. For the two patients who died before discharge, length of hospital stay was defined as the interval from surgery to death; both were retained in all recovery analyses, and in both, the first postoperative bowel movement had been documented before death. C-reactive protein on the first postoperative day was selected a priori as the marker of operative trauma. The acute-phase response to surgery is driven principally by interleukin-6, which is not measured routinely at our institution but induces hepatic CRP synthesis with a predictable lag, so that CRP on the first postoperative day is the earliest routinely available surrogate of the initial inflammatory insult and precedes the infectious complications that dominate CRP later in the postoperative course; this is also the timepoint used in the comparative literature [11]. CRP on the fourth postoperative day is reported descriptively only, because by that time CRP reflects the presence or absence of postoperative complications rather than the magnitude of the original operative insult. CRP is reported in mg/dL according to local laboratory convention; values may be converted to mg/L by multiplying by ten. CME quality was graded prospectively as part of the routine histopathological work-up and not by a review performed for the purposes of this study. Grading was carried out on the fresh, unfixed specimen at macroscopic cut-up, before opening and formalin fixation, by the reporting gastrointestinal pathologist of our institute; for the present analysis, the grades were extracted retrospectively from the original histopathology reports, and no specimen or photographic re-review was undertaken. The Benz–Tannapfel classification stratifies right hemicolectomy specimens according to the extent of mesocolic tissue removed, from type 0, the most radical resection, in which the mesocolic envelope is intact and the central tissue completely removed, to type III, the least radical, and sub-classifies each type by the plane of surgery according to West [12]. Only types 0, 1, and 2 were observed in the present cohort, and a single specimen (1 of 89, 1.1%) was graded 2; grades 1 and 2 were therefore combined, so that the analyzed contrast was between optimal (grade 0) and non-optimal (grade 1 or 2) mesocolic resection. Resection margins were assessed on the same specimens; the radial (mesocolic) margin was documented in the histopathology reports against a 10 mm threshold rather than as an exact measurement and can therefore be reported only dichotomously.

### 2.7. Survival Endpoints

Overall survival (OS) was defined as time from surgery to death from any cause. Disease-free survival (DFS) was defined as time from surgery to recurrence, metastasis, or cancer-related death. Loss to follow-up was treated as censoring and survival was administratively censored at 36 months. Within this observation period, the median follow-up was 36 months in the open and robotic groups and 34.5 (Q1–Q3: 7.9–36.0) months in the laparoscopic group.

### 2.8. Statistical Analysis

As this is a retrospective study, no formal a priori sample size calculation was performed. Instead, we included all patients that fulfilled the inclusion criteria and underwent elective right colectomy for histologically confirmed colon cancer between July 2018 and December 2022 at the University Clinic for General and Visceral Surgery, Medical School OWL, Bielefeld University, Campus Lippe, Detmold. For the statistical analysis, patient characteristics were summarized descriptively. Continuous variables are presented as mean (standard deviation) or median (Q1–Q3), while categorical variables are reported as absolute frequencies and percentages. Kaplan–Meier curves were used to assess OS and DFS across the three surgical approaches for colon cancer. Median follow-up was estimated using the reverse Kaplan–Meier method. A complete-case approach was used. There were no missing values for any covariate included in the regression models, for the study endpoints, or for the variables reported descriptively, and CRP was available on postoperative days one and four in all 89 patients (100%); no imputation was therefore required. Five patients did not complete the full 36-month observation period and were censored at the date of last documented contact.

Binary outcomes were analyzed using multivariable logistic regression, whereas continuous outcomes were assessed using multivariable linear regression. Survival outcomes were evaluated using a multivariable proportional hazards model. All models were adjusted for surgical approach, sex, age, body mass index (BMI), ASA score, UICC stage, and tumor size as covariates. Owing to the small number of patients with ASA score I, ASA scores I and II were grouped. Likewise, due to the limited number of cases with UICC stage 0, UICC stages 0 and I were combined. Regression coefficients, odds ratios, and hazard ratios are presented with corresponding 95% confidence intervals (95% CI). All analyses were performed using R (version 4.5.3). Patients were analyzed according to the intended surgical approach; the five patients who required conversion from laparoscopic to open surgery therefore remained in the laparoscopic group for all analyses, and no patient was reassigned to the open group. The proportional hazards assumption was assessed using Schoenfeld residuals; no evidence of a violation was observed for any individual covariate or in the global test [13]. The Cox models contained 11 effective parameters, and the numbers for risk, events, and censored observations underlying each survival model are reported in Supplementary Tables S10 and S11. Postoperative complications were graded according to the Clavien–Dindo classification [14,15]. As this study was designed neither as an equivalence nor as a non-inferiority study, no margin of clinical equivalence was prespecified, and non-significant findings are reported as such rather than as evidence of comparability.

### 2.9. Ethical Approval

The study protocol was reviewed and approved by the Ethics Committee of the Medical Association of Westphalia-Lippe and the Westphalian Wilhelms University Münster (reference number: 2022-324-f-S, 17 June 2022).

## 3. Results

### 3.1. Patients

Eighty-nine patients were included (open n = 30, laparoscopic n = 29, robotic n = 30). Groups were comparable regarding age, sex, BMI (Table 1).

Differences were observed in ASA scores, with the laparoscopic group including fewer patients with ASA III and a higher proportion of ASA II compared to the other groups.

There were also notable differences in UICC stage distribution: 40% of patients in the open group had stage IV disease, compared with 10.3% and 16.7% in the other two groups, respectively.

### 3.2. Postoperative, Short-Term, Histopathological, and Oncological Outcomes

Postoperative, short-term, histopathological, and oncological outcomes are summarized in Table 2. No complications were recorded in 16 of 29 laparoscopic (55.2%), 18 of 30 open (60.0%), and 22 of 30 robotic patients (73.3%). Major complications (Clavien–Dindo grade III or higher) occurred in three laparoscopic (10.3%), five open (16.7%), and three robotic patients (10.0%). Postoperative ileus was documented in nine (31.0%), seven (23.3%), and three patients (10.0%) and wound infection in three (10.3%), seven (23.3%), and one patient (3.3%), respectively. Anastomotic leakage occurred in two patients, both in the robotic group (6.7%), and in none of the laparoscopic or open procedures. Thirty-day mortality comprised two patients, both in the open group (6.7%), as did 30-day readmission (6.7%); ninety-day mortality was 4 of 30 in the open group (13.3%), 1 of 30 in the robotic group (3.3%), and none in the laparoscopic group. Patient-level details of the two in-hospital deaths, including cause, timing, and whether the death was considered surgery-related, are given in Supplementary Table S12.

Conversion to open surgery was required in 5 of 29 laparoscopic procedures (17.2%) and in none of the robotic procedures. In three cases, the documented reason was dense intra-abdominal adhesions; in the remaining two, conversion was recorded in the operative report without an explicitly stated reason. All converted patients were analyzed according to the intended surgical approach and therefore remained in the laparoscopic group for all analyses.

R0 resection was achieved in 87 of 89 patients (97.8%), and both R1 resections occurred in the open group. Advanced pathological stage was more frequent in the open group, with pT4 tumors in 9 of 30 patients (30.0%) compared with 4 of 29 (13.8%) and 4 of 30 (13.3%) in the laparoscopic and robotic groups, and node-positive disease in 21 of 30 (70.0%) compared with 15 of 29 (51.7%) and 11 of 30 (36.7%). Lymphovascular, vascular, and perineural invasion were likewise most frequent in the open group. Characteristics of the 20 patients with UICC stage IV disease, including the site and burden of metastatic disease, operative intent, metastasectomy, residual disease after colectomy, and whether a state of no evidence of disease was achieved, are reported in Supplementary Table S13. These comparisons are descriptive and were not part of the predefined endpoint structure; no formal hypothesis tests were therefore performed, and proportions are reported with their denominators. All longitudinal resection margins were well in excess of the length required for an oncologically adequate right colectomy, the shortest measured margin in the cohort being 9 cm, and the radial (mesocolic) margin was at least 10 mm in 88 of 89 specimens (98.9%). The single specimen with a radial margin below 10 mm was an open pT4a tumor, which was also one of the two R1 resections.

### 3.3. Primary Endpoint

Associations between the surgical approach and time to first postoperative bowel movement were analyzed. The first postoperative bowel movement occurred earlier in the robotic group (3.9 days, SD 1.9), followed by the laparoscopic group (4.21 days, SD 2.32) and the open group (5.13 days, SD 1.68) (Table 3).

### 3.4. Secondary Endpoints

In the minimally invasive groups, the patients were discharged earlier (laparoscopic 9.14 days and robotic 9.93 days, respectively) than in the open group (10.1 days). The highest lymph node counts were observed in the open and laparoscopic right colectomy groups, followed by the robotic group (Table 3).

Grade 0 CME quality predominated in all groups. The highest proportion of Grade 0 resections was observed in the robotic-assisted right colectomy group (80%), followed by the laparoscopic (72.4%) and open right colectomy group (66.7%) (Table 3).

On postoperative day one, the lowest CRP level was observed in the robotic group (5.5 mg/dL, SD 2.79), followed by the laparoscopic group (5.96 mg/dL, SD 3.70), and the highest levels were in the open group (8.33 mg/dL, SD 4.99) (Table 3).

Operative duration was shorter in the open right colectomy group compared with both minimally invasive groups, with a mean operative time of 137 min (SD 45.1) versus 181 min (SD 41.7) in laparoscopic group and 193 min (SD 37.9) in the robotic group (Table 3).

### 3.5. Regression Analyses

Postoperative bowel movement occurred earlier in both minimally invasive groups compared with the open group, although the differences were not statistically significant (Table 4, Supplementary Tables S1 and S2). In the robotic group, bowel function returned on average 1.1 days earlier than in the open group (mean difference −1.1 days, 95% CI: [−2.4, 0.10]; *p* = 0.070). In the laparoscopic group, bowel movement occurred 0.55 days earlier than in the open group (mean difference: −0.55 days; 95% CI: [−1.7, 0.63]; *p* = 0.354). After adjustment, there were no statistically significant differences in length of hospital stay between the surgical techniques (robotic vs. open: 0.47 days, 95% CI: [−2.2, 3.2], *p* = 0.734; laparoscopic vs. open: 0.33 days, 95% CI: [−2.2, 2.9], *p* = 0.798).

Oncological quality surrogate parameters, including lymph node yield and CME quality, were not statistically significantly associated with surgical approach (Table 4, Supplementary Tables S5 and S6).

Postoperative CRP on day 1 showed a tendency toward lower levels in both minimally invasive groups compared with the open group (robotic vs. open: −2.0 mg/dL, 95% CI: [−4.4, 0.47], *p* = 0.111; laparoscopic vs. open: −2.0 mg/dL, 95% CI: [−4.3, 0.29], *p* = 0.086). Operative time was longer for both robotic and laparoscopic procedures compared with open surgery (robotic vs. open: +50 min, 95% CI: [25, 75], *p* < 0.001; laparoscopic vs. open: +43 min, 95% CI: [19, 67], *p* < 0.001) (Supplementary Tables S3 and S4). Regression diagnostic plots for the two recovery outcomes, comprising residuals versus fitted values and normal Q–Q plots, are provided in Supplementary Figures S2 and S3; these did not indicate substantial deviations from the assumptions of linearity, homoscedasticity, or approximate normality of the residuals.

### 3.6. Disease-Free Survival

**Figure 1:**
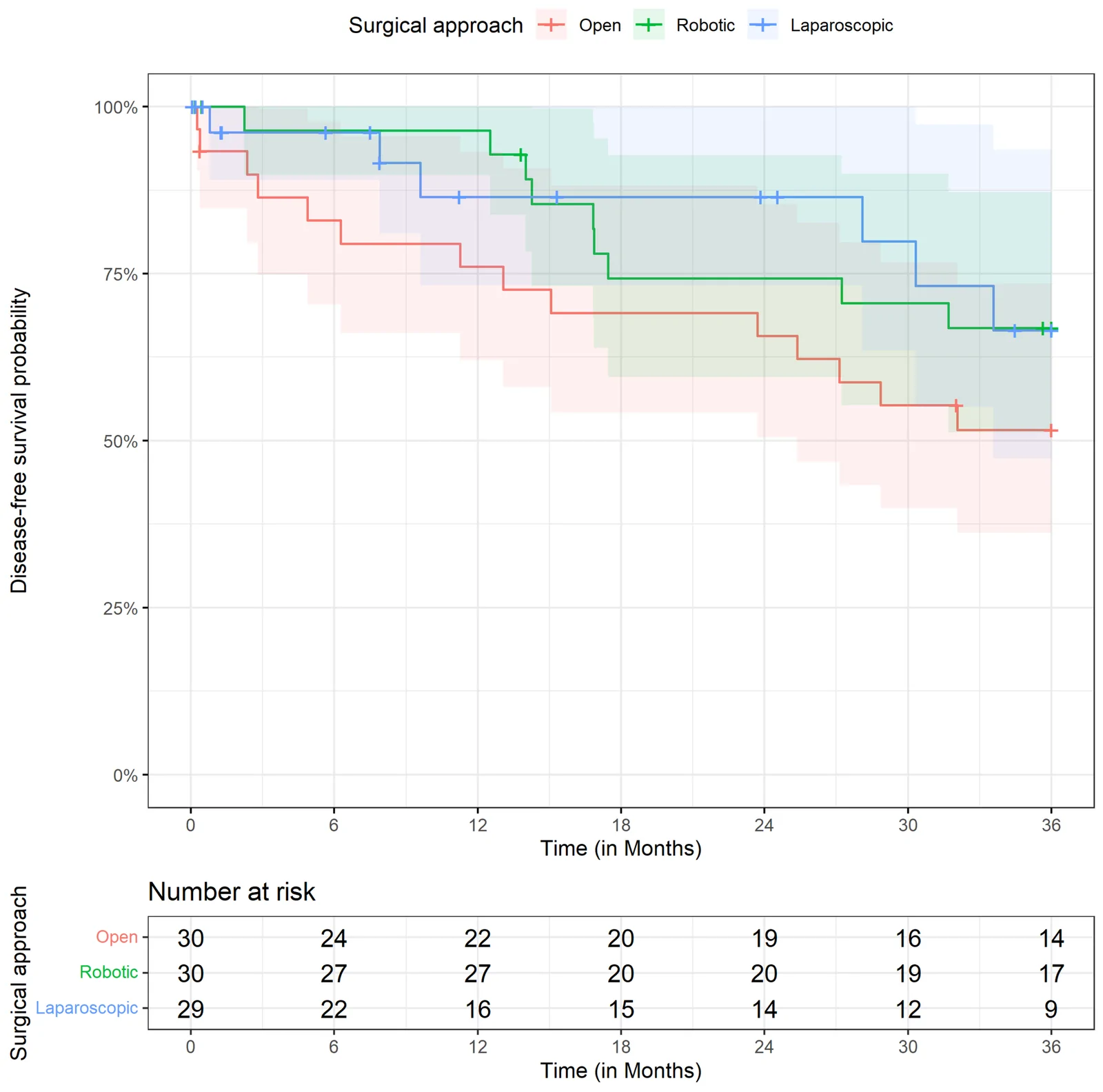
Disease-free survival in open, laparoscopic, and robotic right colectomy groups.

DFS was analyzed using Kaplan–Meier curves and administrative censoring at 36 months (Figure 1). DFS was numerically higher in the minimally invasive groups compared with open surgery; the robotic and laparoscopic estimates were of similar magnitude, but the confidence intervals were wide and overlapping. The estimated DFS probabilities were 51.6% (95% CI: [36.2, 73.5]) for open surgery, 66.9% (95% CI: [51.3, 87.2]) for robotic surgery, and 66.5% (95% CI: [47.3, 93.6]) for laparoscopic surgery. However, compared with open surgery, hazard ratios indicated no statistically significant differences in DFS for robotic (HR 0.89, 95% CI: [0.32, 2.53]; *p* = 0.833) or laparoscopic surgery (HR 0.88, 95% CI: [0.31, 2.52]; *p* = 0.814) (Table 4, Supplementary Table S8). A sensitivity analysis restricted to patients with UICC stages I–III, excluding the 20 patients with stage IV disease, was consistent with the primary analysis and did not change the conclusions (robotic vs. open HR 0.71, 95% CI: [0.14, 3.63]; laparoscopic vs. open HR 0.57, 95% CI: [0.11, 3.08]; Supplementary Table S9 and Supplementary Figure S1).

### 3.7. Overall Survival

**Figure 2:**
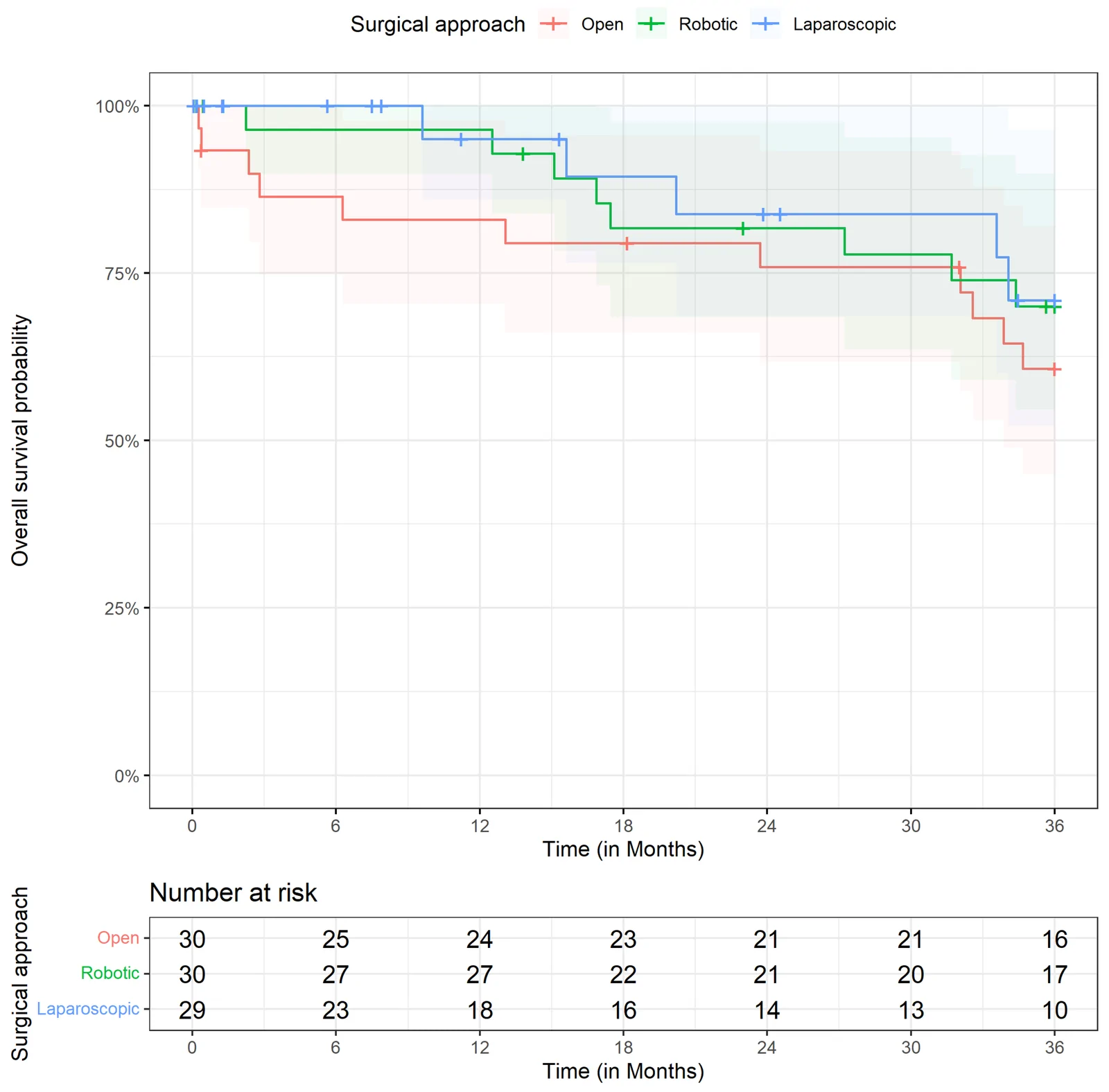
OS in open, laparoscopic, and robotic right colectomy groups.

OS was numerically higher in both minimally invasive groups compared with open surgery; as for DFS, the robotic and laparoscopic estimates were of similar magnitude with wide, overlapping confidence intervals (Figure 2). The estimated OS probabilities were 60.7% (95% CI: [45.0, 82.0]) for open surgery, 70.0% (95% CI: [54.6, 89.8]) for robotic surgery, and 70.9% (95% CI: [52.2, 96.4]) for laparoscopic surgery. However, OS did not differ statistically significantly between surgical approaches. Compared with open surgery, the hazard ratios were 1.16 (95% CI: [0.39, 3.43]; *p* = 0.786) for robotic surgery and 1.01 (95% CI: [0.31, 3.23]; *p* = 0.993) for laparoscopic surgery. Compared with UICC stage 0/I, higher UICC stages were associated with an increased hazard of death, with only stage IV showing a statistically significant association with worse overall survival (HR 17.3, 95% CI [1.92, 156]; *p* = 0.011); the width of this interval reflects the small number of events and the estimate should not be read as a precise measure of effect (Table 4, Supplementary Table S7). Detailed numbers for risk, events, and survival probabilities over time for overall survival and disease-free survival, stratified by group, are provided in Supplementary Tables S10 and S11.

## 4. Discussion

Minimally invasive right colectomy for colon cancer has gained widespread acceptance over the last two decades, yet direct comparisons between open, laparoscopic, and robotic-assisted approaches remain limited [6,7]. In the present study, postoperative recovery was analyzed as the primary endpoint, with surgical quality and oncological outcomes evaluated as secondary measures. Our findings show numerically faster postoperative recovery following minimally invasive surgery, and no statistically significant difference in oncological resection quality could be demonstrated across the approaches. Unadjusted survival estimates were numerically higher after minimally invasive surgery; however, none of these differences remained statistically significant after multivariable adjustment, and no conclusion of a survival benefit can be drawn. Time to first postoperative bowel movement and length of hospital stay were numerically shorter after minimally invasive surgery in our cohort, with the shortest medians observed after robotic-assisted surgery, although neither difference reached statistical significance after adjustment. This direction is consistent with the existing literature comparing minimally invasive and open colectomy. Large, randomized trials and meta-analyses have demonstrated earlier gastrointestinal recovery and shorter hospitalization after laparoscopic colectomy compared with open surgery [7,16,17]. Comparisons between laparoscopic and robotic right colectomy are more heterogeneous. Several recent systematic reviews report modest reductions in time to bowel function and length of stay following robotic surgery, although absolute differences are often small and influenced by institutional pathways [4,5,6]. The relatively long hospital stays observed in our cohort across all groups probably reflect health system-specific discharge practices rather than surgical approach alone, which may explain the absence of statistically significant differences.

We could not demonstrate a difference in lymph node yield or CME quality between the three surgical approaches, although the precision of these comparisons was limited. This is in keeping with previous studies reporting similar oncological quality for open and minimally invasive right colectomy when complete mesocolic excision and central vascular ligation are performed [12,16]. Reported lymph node yields in robotic and laparoscopic CME series typically range between 25 and 36 nodes, comparable to our results [2,18]. The highest proportion of optimal CME quality was observed in the robotic-assisted group, but this difference was not statistically significant (adjusted odds ratio for a non-optimal specimen, robotic vs. open, 0.58, 95% CI 0.12–2.56) and should not be over-interpreted. A similar observation has been reported by others, who suggest that enhanced visualization and instrument articulation may facilitate precise mesocolic plane dissection in robotic surgery [19]. However, current evidence does not support oncological superiority of robotic surgery based on specimen quality alone.

A reduction in CRP levels on postoperative day one was observed after laparoscopic and robotic-assisted surgery compared with open surgery. This finding is biologically plausible and consistent with prior studies demonstrating lower systemic inflammatory response after minimally invasive colorectal surgery [11,20]. Reduced inflammatory response has been associated with fewer postoperative complications and faster functional recovery, although causality remains difficult to establish. Direct comparisons of inflammatory markers between robotic and laparoscopic colectomy suggest similar or slightly reduced inflammatory responses following robotic procedures [20]. The direction of our CRP findings is consistent with the concept of reduced surgical trauma after minimally invasive surgery, but the difference did not reach statistical significance after adjustment (robotic vs. open −2.0 mg/dL, 95% CI −4.4 to 0.47; laparoscopic vs. open −2.0 mg/dL, 95% CI −4.3 to 0.29) and should not be interpreted as an established effect. Both confidence intervals included zero, and this study was not powered to detect a difference of this magnitude. Even with randomised evidence in which an early difference in interleukin-6 and CRP is demonstrable, results do not translate into a difference in infectious complication rates [11]; a lower early CRP is therefore a surrogate of uncertain clinical consequence. CRP on postoperative day four, which is dominated by the presence or absence of complications rather than by the operative insult, was closely similar across the three groups (mean 8.07, 8.55, and 7.90 mg/dL in the laparoscopic, open, and robotic groups).

Operative time was statistically significantly longer in minimally invasive procedures, particularly in the robotic-assisted group. This finding is consistent with many comparative studies and meta-analyses [20,21,22,23,24]. Prolonged operative time is commonly attributed to increased procedural complexity and learning curve effects. The robotic procedures reported here were performed during the learning curve of the operating surgeon for this specific procedure and not after a plateau had been reached; operative time in the robotic group did not decline over the study period, and we have analyzed this learning curve formally in a separate report [21]. The operative times presented here should therefore be read as those of an early robotic programme rather than as the steady-state performance of a mature one, which works against the robotic approach in this comparison. It should also be noted that intracorporeal anastomotic reconstruction differed between laparoscopic (stapled) and robotic (hand-sewn) procedures, which may have contributed to differences in operative time. Importantly, several reports demonstrate a reduction in operative time with increasing robotic experience and standardized workflows [5,25,26]. Conversion to open surgery was required in 5 of 29 laparoscopic procedures (17.2%) and in none of the robotic procedures; dense intra-abdominal adhesions were the documented reason in three of these five cases. Previous studies have reported lower conversion rates in robotic colectomy compared with laparoscopic surgery, particularly in technically demanding cases [24,25]. Conversion has been consistently associated with worse short-term outcomes and prolonged hospitalization [23,24,25], which may indirectly influence recovery metrics.

Both anastomotic leaks in this cohort occurred in the robotic group, in which reconstruction was performed as a hand-sewn intracorporeal anastomosis, whereas all laparoscopic anastomoses were stapled. With two events among 89 patients, no meaningful comparison of leak rates between the approaches is possible, and the difference may equally reflect chance or the early phase of the robotic programme; one of these two patients accounted for the single death in the robotic group within 90 days. It is nevertheless relevant that the comparison between the two minimally invasive groups is not one of access platforms alone. Reconstruction technique was a deterministic function of the approach in this cohort and is therefore perfectly confounded with it, for operative time and return of bowel function as well as for anastomotic outcome, and we no longer attribute the earlier return of bowel function in the robotic group to the platform itself.

The prolonged operative time observed in both minimally invasive groups also has direct economic implications, as operating room time is one of the principal cost drivers in colorectal surgery. Beyond this, robotic surgery carries substantial additional costs arising from platform acquisition, maintenance contracts, and single-use instruments. A recent systematic review and meta-analysis of comparative studies confirmed significantly higher total and operative costs for robotic compared with laparoscopic colorectal resection, although with very high statistical heterogeneity, reflecting the marked variation in health-care systems, case volumes, and costing methodologies between studies [27]. In a national inpatient analysis restricted to right colectomy, mean hospital costs were USD 12,516 for the laparoscopic and USD 15,027 for the robotic approach, corresponding to an increase of approximately 20% [28]. Since postoperative recovery, surgical quality, and three-year survival did not differ significantly between approaches in our cohort, these additional costs are currently not offset by a demonstrable clinical benefit in this indication. This is particularly relevant in the German setting, where no dedicated reimbursement category for robotic-assisted surgery exists and the additional expenditure must be absorbed by the institution. Whether increasing case volume, shorter operative times with growing experience and declining consumable prices will alter this balance remains to be determined in prospective health–economic analyses.

Unadjusted overall and disease-free survival estimates were numerically higher in the minimally invasive groups. After multivariable adjustment, no statistically significant association between surgical approach and survival remained. This finding is in line with the prevailing literature, in which no consistent survival difference among open, laparoscopic, and robotic colectomy has been demonstrated [23,24]. Given the limited statistical power, the wide confidence intervals, and the marked baseline imbalance in tumour stage between groups, the unadjusted differences are most likely to reflect patient selection rather than any effect of the surgical approach. These findings should be interpreted cautiously. Most comparative studies and meta-analyses report similar DFS and OS between robotic and laparoscopic right colectomy when adjusted for confounding factors [23,24]. A recent systematic review focusing on CME-based right colectomy found no consistent survival advantage for robotic over laparoscopic approaches [29]. In our study, the limited statistical power argues against definitive conclusions regarding oncological superiority. Although unadjusted analyses suggested improved survival in minimally invasive groups, these differences were not confirmed after multivariable adjustment. This indicates that the observed survival differences are likely to have been influenced by baseline characteristics rather than surgical approach itself.

Our findings are consistent with current literature suggesting that long-term oncological outcomes are primarily driven by tumour biology rather than operative technique. The present analysis detected no statistically significant independent association between the surgical approach and postoperative recovery or long-term oncological outcomes. This should not be read as evidence of equivalence; the study was not designed or powered for that purpose, and clinically important differences cannot be excluded on the basis of these data. Observed differences in unadjusted analyses appear to have been driven by patient selection and tumour stage rather than surgical technique itself.

The retrospective design, limited sample size, and sequential implementation of surgical techniques limit the interpretability of these findings. Despite the relatively small sample size, multivariable regression models were considered the most appropriate analytical approach, as they enabled direct adjustment for clinically relevant covariates while making efficient use of the available data. When appropriately specified, conventional multivariable regression provides statistically efficient and consistent effect estimates and may be preferable to propensity score-based methods in smaller samples, where estimation of the propensity score itself may introduce additional uncertainty. Nevertheless, the limited sample size may have reduced the precision of the adjusted estimates and limited the statistical power to detect clinically meaningful differences. Furthermore, residual confounding cannot be excluded. A further limitation is the small number of patients who died before discharge. In these patients, time to death was used as the endpoint for length of stay, which may introduce bias when comparing postoperative length of stay between groups. A competing-risk or time-to-discharge analysis would be preferable; however, the limited number of deaths in our cohort did not allow for a sufficiently robust analysis. An additional limitation of this study is the potential for temporal bias related to the sequential introduction of robotic surgery. As robotic procedures were implemented later than open surgery, differences in outcomes may not solely reflect the surgical approach itself but may also be influenced by contemporaneous improvements in perioperative care, increasing surgical experience, refinement of institutional pathways, and the implementation of enhanced recovery after surgery (ERAS) protocols. Therefore, the influence of temporal changes cannot be completely excluded. In addition, health-economic endpoints were not assessed. Cost data were not part of the predefined study protocol and could not be reliably reconstructed retrospectively, as robotic, laparoscopic, and open right colectomy are reimbursed within the same German DRG and patient-level cost accounting was not consistently available over the entire study period. No conclusions regarding the cost-effectiveness of the three approaches can therefore be drawn from our data.

Two further limitations concern the assessment of complete mesocolic excision. Because grading was embedded in routine clinical reporting, the assessing pathologist was aware of the operative approach, which was stated on the pathology request form; therefore, assessment was not blinded. Each specimen was graded once by a single pathologist, and interobserver agreement could therefore not be evaluated. In addition, only 24 non-optimal specimens were observed in the whole cohort, and the adjusted model contained a substantial number of covariates relative to that number of events; the confidence intervals around the odds ratios for CME quality are correspondingly wide, and these estimates are best read as showing no detectable difference in specimen quality between the three approaches rather than as precise effect estimates.

A further limitation concerns systemic therapy. All patients with node-positive disease were discussed at the institutional multidisciplinary tumour board and referred to medical oncology for consideration of adjuvant chemotherapy in accordance with national guidelines [8]. Adjuvant treatment is, however, delivered in the outpatient setting and frequently by practices outside our institution, so that from the records available to a surgical department, it is not possible to establish reliably whether treatment was actually started, which regimen was used, whether dose reductions or premature discontinuation occurred, or what toxicity was encountered. We have therefore not reported adjuvant chemotherapy as a variable, since a simple received-or-not indicator would be neither complete nor accurate in this setting. Systemic therapy is consequently an uncontrolled factor in the comparison of survival between the groups, and its contribution cannot be separated from that of tumour stage in a cohort of this size, which is a further reason why no conclusion about survival is drawn from these data.

The sequential introduction of the three techniques deserves more than a general caveat, and we therefore report the relevant distributions in the Supplementary Material. The robotic programme began in February 2021 and the last open resection in this cohort was performed in November 2021, so that robotic and open procedures overlap in only a narrow window, and 23 of 30 robotic cases (76.7%) were performed in the final calendar year (Supplementary Table S15). Surgical approach and calendar era can therefore not be separated in this cohort, and we cannot exclude that part of the numerically faster recovery observed in the robotic group reflects secular improvement in perioperative care rather than the access platform. Twelve surgeons contributed to the open group, seven to the laparoscopic group, and four to the robotic group (Supplementary Table S14). All operators were formally designated colorectal surgeons within a certified colorectal cancer centre and met the required minimum annual volume of oncological resections throughout the study period, so that the wider dispersion of the open group across operators does not represent a mix of experienced and inexperienced surgeons. The minimally invasive procedures were nevertheless concentrated in a single surgeon, who performed 25 of 30 robotic and 17 of 29 laparoscopic operations; the laparoscopic-versus-robotic comparison is thus largely a within-surgeon comparison, whereas the open-versus-minimally-invasive comparison is distributed across many operators and is correspondingly more vulnerable to surgeon effects. Calendar year, operating surgeon, and anastomotic technique were not entered into the multivariable models because each is almost or entirely collinear with the exposure of interest; including them would have produced unstable estimates and a false impression of control rather than genuine adjustment. Separating these effects would require a prospective design in which the approaches are performed contemporaneously and the reconstruction technique is standardized across platforms.

This study was designed neither as an equivalence nor as a non-inferiority study, and no formal margin of clinical equivalence was prespecified. The absence of a statistically significant difference between the approaches must therefore not be interpreted as evidence that the approaches are equivalent. With 89 patients distributed across three groups, the confidence intervals around the adjusted estimates are wide and remain compatible with clinically meaningful differences in either direction. For example, the adjusted hazard ratio for disease-free survival in the robotic group was 0.89 with a 95% confidence interval of 0.32 to 2.53, an interval compatible both with a substantial reduction and with a more than two-fold increase in the hazard of recurrence or death. We did not perform a post hoc power calculation, since observed power is essentially a one-to-one transformation of the observed *p*-value and adds nothing to the effect estimate and confidence interval already reported [30]. Our findings should accordingly be read as a description of a single-centre cohort in which no independent effect of the surgical approach could be demonstrated, and not as a demonstration that no such effect exists.

Finally, this is a retrospective single-centre study conducted in a high-volume certified colorectal cancer centre, and its external validity is correspondingly limited. The case mix, perioperative pathways, and operator experience described here may not be representative of lower-volume institutions or of other health-care systems, and the absolute values reported—in particular, the length of hospital stay—are strongly influenced by national discharge practice. Confirmation in prospective multicentre studies, ideally randomised and with contemporaneous performance of the three approaches, is required before these findings can be generalised. Overall, the study provides real-world comparative data across all three approaches within a standardized institutional framework.

## 5. Conclusions

In conclusion, within this single-centre cohort, no statistically significant independent association between surgical approach and postoperative recovery or three-year oncological outcomes was detected once baseline characteristics were considered. The differences seen in unadjusted analyses appear to reflect patient selection and tumor stage rather than the surgical approach itself. However, given the limited sample size and the potential for residual confounding, clinically relevant differences cannot be excluded. The choice of approach should be guided by patient-specific factors, surgeon expertise, and available resources. Larger prospective studies are warranted to confirm these findings.

## Figures and Tables

**Table 1:** Patient characteristics.

	Laparoscopic (N = 29)	Open (N = 30)	Robotic (N = 30)	Overall (N = 89)
Sex				
Females	12 (41.4%)	11 (36.7%)	15 (50.0%)	38 (42.7%)
Males	17 (58.6%)	19 (63.3%)	15 (50.0%)	51 (57.3%)
Age (in years)				
Mean (SD)	70.9 (12.3)	74.8 (10.7)	71.9 (12.2)	72.6 (11.8)
Median [Q1, Q3]	73.0 [63.0, 81.0]	78.0 [66.3, 82.0]	76.5 [62.5, 81.8]	75.0 [64.0, 82.0]
BMI (kg/m^2^)				
Mean (SD)	26.1 (3.80)	27.4 (4.92)	27.7 (5.04)	27.1 (4.63)
Median [Q1, Q3]	25.5 [23.5, 27.8]	27.0 [24.1, 29.4]	26.7 [24.1, 30.5]	26.2 [24.1, 28.7]
Comorbidities	21 (72.4%)	23 (76.7%)	22 (73.3%)	66 (74.2%)
Previous surgeries	12 (41.4%)	7 (23.3%)	16 (53.3%)	35 (39.3%)
ASA score				
I	1 (3.4%)	0 (0%)	0 (0%)	1 (1.1%)
II	15 (51.7%)	8 (26.7%)	8 (26.7%)	31 (34.8%)
III	13 (44.8%)	21 (70.0%)	20 (66.7%)	54 (60.7%)
IV	0 (0%)	1 (3.3%)	2 (6.7%)	3 (3.4%)
UICC Stage				
0	1 (3.4%)	0 (0%)	4 (13.3%)	5 (5.6%)
I	4 (13.8%)	3 (10.0%)	10 (33.3%)	17 (19.1%)
II	10 (34.5%)	5 (16.7%)	6 (20.0%)	21 (23.6%)
III	11 (37.9%)	10 (33.3%)	5 (16.7%)	26 (29.2%)
IV	3 (10.3%)	12 (40.0%)	5 (16.7%)	20 (22.5%)
Tumor location				
Ascending colon	17 (58.6%)	19 (63.3%)	21 (70.0%)	57 (64.0%)
Right colonic flexure	1 (3.4%)	1 (3.3%)	1 (3.3%)	3 (3.4%)
Cecum	11 (37.9%)	10 (33.3%)	8 (26.7%)	29 (32.6%)
Tumor size (in mm)				
Mean (SD)	44.9 (17.1)	43.9 (13.9)	45.2 (25.2)	44.7 (19.2)
Median [Q1, Q3]	45.0 [36.0, 56.0]	43.0 [38.0, 48.0]	42.5 [24.0, 64.0]	43.0 [33.0, 55.0]

**Table 2:** Post-operative, Short-Term, Histopathological, and Oncological Outcomes.

	Laparoscopic (N = 29)	Open (N = 30)	Robotic (N = 30)	Overall (N = 89)
Post-operative and short-term outcomes				
30-day mortality				
Yes	0 (0%)	2 (6.7%)	0 (0%)	2 (2.2%)
Lost to follow up	2 (6.9%)	1 (3.3%)	2 (6.7%)	5 (5.6%)
90-day mortality	0 (0%)	4 (13.3%)	1 (3.3%)	5 (5.6%)
30-day readmission	0 (0%)	2 (6.7%)	0 (0%)	2 (2.2%)
Anastomotic leakage	0 (0%)	0 (0%)	2 (6.7%)	2 (2.2%)
Clavien–Dindo				
0	16 (55.2%)	18 (60.0%)	22 (73.3%)	56 (62.9%)
I	8 (27.6%)	7 (23.3%)	5 (16.7%)	20 (22.5%)
II	2 (6.9%)	0 (0%)	0 (0%)	2 (2.2%)
III	2 (6.9%)	3 (10.0%)	1 (3.3%)	6 (6.7%)
IV	1 (3.4%)	0 (0%)	2 (6.7%)	3 (3.4%)
V	0 (0%)	2 (6.7%)	0 (0%)	2 (2.2%)
Postoperative ileus	9 (31.0%)	7 (23.3%)	3 (10.0%)	19 (21.3%)
Wound infection	3 (10.3%)	7 (23.3%)	1 (3.3%)	11 (12.4%)
Pulmonary complications	0 (0%)	1 (3.3%)	1 (3.3%)	2 (2.2%)
Cardiovascular complications	1 (3.4%)	0 (0%)	0 (0%)	1 (1.1%)
Conversion	5 (17.2%)	0 (0%)	0 (0%)	5 (5.6%)
Histopathological and oncological outcomes				
Pathological T stage (pT)				
pT1	2 (6.9%)	2 (6.7%)	8 (26.7%)	12 (13.5%)
pT2	3 (10.3%)	4 (13.3%)	6 (20.0%)	13 (14.6%)
pT3	20 (69.0%)	15 (50.0%)	12 (40.0%)	47 (52.8%)
pT4	4 (13.8%)	9 (30.0%)	4 (13.3%)	17 (19.1%)
Pathological N stage (pN)				
pN0	14 (48.3%)	9 (30.0%)	19 (63.3%)	42 (47.2%)
pN1	10 (34.5%)	12 (40.0%)	5 (16.7%)	27 (30.3%)
pN2	5 (17.2%)	9 (30.0%)	6 (20.0%)	20 (22.5%)
Distant metastasis (M)				
M0	27 (93.1%)	18 (60.0%)	25 (83.3%)	70 (78.7%)
M1	2 (6.9%)	12 (40.0%)	5 (16.7%)	19 (21.3%)
Tumor grading (G)				
0	0 (0%)	0 (0%)	1 (3.3%)	1 (1.1%)
1	1 (3.4%)	0 (0%)	6 (20.0%)	7 (7.9%)
2	25 (86.2%)	25 (83.3%)	19 (63.3%)	69 (77.5%)
3	3 (10.3%)	5 (16.7%)	4 (13.3%)	12 (13.5%)
Resection status (R)				
R0	29 (100%)	28 (93.3%)	30 (100%)	87 (97.8%)
R1	0 (0%)	2 (6.7%)	0 (0%)	2 (2.2%)
Proximal margin (cm), median [Q1, Q3]	19.0 [15.0, 22.0]	17.0 [16.0, 19.0]	15.0 [14.0, 18.0]	17.0 [15.0, 19.0]
Distal margin (cm), median [Q1, Q3]	16.0 [13.0, 20.0]	16.0 [14.6, 17.8]	18.0 [15.0, 21.0]	16.0 [14.0, 19.5]
Radial (mesocolic) margin ≥ 10 mm	29 (100%)	29 (96.7%)	30 (100%)	88 (98.9%)
Lymphovascular invasion (L)	12 (41.4%)	19 (63.3%)	6 (20.0%)	37 (41.6%)
Vascular invasion (V)	2 (6.9%)	9 (30.0%)	4 (13.3%)	15 (16.9%)
Perineural invasion (Pn)	1 (3.4%)	5 (16.7%)	3 (10.0%)	9 (10.1%)

**Table 3:** Primary and secondary endpoints according to surgical approach.

	Laparoscopic (N = 29)	Open (N = 30)	Robotic (N = 30)	Overall (N = 89)
Op duration (in minutes)				
Mean (SD)	181 (41.7)	137 (45.1)	193 (37.9)	170 (47.8)
Median [Q1, Q3]	174 [153, 211]	131 [111, 151]	189 [164, 214]	163 [137, 202]
Length of hospital stay				
Mean (SD)	9.14 (4.37)	10.1 (3.88)	9.93 (5.88)	9.74 (4.76)
Median [Q1, Q3]	8.00 [6.00, 11.0]	8.50 [7.00, 12.8]	8.00 [6.00, 11.0]	8.00 [6.00, 12.0]
Lymph nodes yield				
Mean (SD)	34.2 (9.57)	34.2 (11.3)	33.6 (12.1)	34.0 (10.9)
Median [Q1, Q3]	32.0 [27.0, 40.0]	33.5 [26.0, 42.5]	32.0 [24.3, 43.5]	33.0 [26.0, 43.0]
CME quality				
0	21 (72.4%)	20 (66.7%)	24 (80.0%)	65 (73.0%)
1 & 2	8 (27.6%)	10 (33.3%)	6 (20.0%)	24 (27.0%)
Postoperative bowel movement (in days)				
Mean (SD)	4.21 (2.32)	5.13 (1.68)	3.90 (1.90)	4.42 (2.03)
Median [Q1, Q3]	4.00 [3.00, 4.00]	5.00 [4.00, 6.00]	3.50 [2.25, 5.00]	4.00 [3.00, 5.00]
Postoperative CRP—day 1 (mg/dL)				
Mean (SD)	5.96 (3.70)	8.33 (4.99)	5.50 (2.79)	6.60 (4.09)
Median [Q1, Q3]	5.10 [3.10, 7.50]	7.10 [5.30, 10.3]	4.90 [3.33, 7.40]	5.60 [3.90, 8.40]
Postoperative CRP—day 4 (mg/dL)				
Mean (SD)	8.07 (7.41)	8.55 (6.03)	7.90 (4.95)	8.17 (6.13)
Median [Q1, Q3]	6.10 [3.80, 10.6]	7.85 [3.68, 11.4]	6.30 [4.63, 9.60]	6.50 [3.90, 10.6]
Reoperation	2 (6.9%)	3 (10.0%)	2 (6.7%)	7 (7.9%)
Death during hospital stay	0 (0%)	2 (6.7%)	0 (0%)	2 (2.2%)

**Table 4:** Multivariable regression analyses of the primary and secondary outcomes, adjusted for surgical approach, age, sex, body mass index, ASA score, UICC stage, and tumor size. For the surgical approach, open surgery was used as the reference category. CI, confidence interval; HR, hazard ratio. In the logistic regression model for CME quality the modelled event is a non-optimal specimen (Benz–Tannapfel grade 1 or 2); an odds ratio below 1 therefore denotes lower odds of a non-optimal specimen than after open surgery.

	Mean Difference	95% CI	*p*-Value
Postoperative bowel movement (in days)			
Robotic vs. Open	−1.1	[−2.4, 0.10]	0.070
Laparoscopic vs. Open	−0.55	[−1.7, 0.63]	0.354
Length of hospital stay			
Robotic vs. Open	0.47	[−2.2, 3.2]	0.734
Laparoscopic vs. Open	0.33	[−2.2, 2.9]	0.798
Postoperative CRP—day 1 (mg/dL)			
Robotic vs. Open	−2.0	[−4.4, 0.47]	0.111
Laparoscopic vs. Open	−2.0	[−4.3, 0.29]	0.086
Op duration (in minutes)			
Robotic vs. Open	50	[25, 75]	<0.001
Laparoscopic vs. Open	43	[19, 67]	<0.001
Lymph nodes yield			
Robotic vs. Open	−1.9	[−8.2, 4.4]	0.555
Laparoscopic vs. Open	−3.8	[−9.7, 2.2]	0.214
	Odds Ratio	95% CI	*p*-value
CME quality			
Robotic vs. Open	0.58	[0.12, 2.56]	0.476
Laparoscopic vs. Open	1.16	[0.31, 4.49]	0.825
	Hazard Ratio	95% CI	*p*-value
Overall survival			
Robotic vs. Open	1.16	[0.39, 3.43]	0.786
Laparoscopic vs. Open	1.01	[0.31, 3.23]	0.993
Disease-free survival			
Robotic vs. Open	0.89	[0.32, 2.53]	0.833
Laparoscopic vs. Open	0.88	[0.31, 2.52]	0.814

## Data Availability

The data presented in this study are available on reasonable request from the corresponding author. The data are not publicly available due to privacy and ethical restrictions concerning patient information.

## Supplementary Materials

The following supporting information can be downloaded at: https://www.mdpi.com/article/10.3390/cancers18172769/s1. Table S1: multivariable linear regression for postoperative bowel movement; Table S2: multivariable linear regression for length of hospital stay; Table S3: multivariable linear regression for C-reactive protein on postoperative day 1; Table S4: multivariable linear regression of the operation; Table S5: multivariable linear regression for lymph nodes yield; Table S6: multivariable logistic regression for complete mesocolic excision quality; Table S7: multivariable Cox proportional hazards model for overall survival; Table S8: multivariable Cox proportional hazards model for disease-free survival; Table S9: multivariable Cox proportional hazards model for disease-free survival restricted to UICC stages I–III; Table S10: Kaplan–Meier estimates of overall survival including numbers for risk, events, and censoring over time; Table S11: Kaplan–Meier estimates of disease-free survival including numbers for risk, events, and censoring over time; Table S12: patient-level details of the in-hospital deaths; Table S13: characteristics of patients with UICC stage IV disease; Table S14: distribution of procedures by year and surgeon; Table S15: distribution of procedures by year; Figure S1: disease-free survival restricted to UICC stages I–III; Figure S2: regression diagnostics for postoperative bowel movement; Figure S3: regression diagnostics for length of hospital stay.
