# Supplementary material for: Comparative Outcomes of Open, Laparoscopic, and Robotic Right Colectomy for Colon Cancer: Postoperative Recovery, Surgical Quality, and 3-Year Survival—A Monocentric Retrospective Study
Source: Cancers (Basel). 2026 Aug 26;18(17):2769. doi: 10.3390/cancers18172769 (PMC13565720; doi:10.3390/cancers18172769)
Supplement: Supplementary file 1 [file cancers-18-02769-s001.zip › cancers-4454042-supplementary.pdf]

Supplementary Materials

Postoperative bowel movement (in days)

Table S1: Multivariable linear regression for postoperative bowel movement (in days), adjusting for surgical approach, age, sex, BMI, UICC-Stage, ASA Score and tumor size

Characteristic	Mean difference	95% CI[1]	p-value
**Surgical approach**			
Robotic vs Open	-1.1	[-2.4, 0.10]	0.070
Laparoscopic vs. Open	-0.55	[-1.7, 0.62]	0.354
**Sex**			
Males vs. Females	0.07	[-0.90, 1.0]	0.893
**Age**	0.02	[-0.03, 0.07]	0.410
**BMI**	0.03	[-0.07, 0.13]	0.601
**UICC-Stage**			
Stage II vs. Stage 0/I	-1.1	[-2.6, 0.48]	0.176
Stage III vs. Stage 0/I	-0.47	[-2.0, 1.1]	0.547
Stage IV vs. Stage 0/I	0.31	[-1.3, 1.9]	0.702
**ASA Score**			
III vs. I/II	-0.08	[-1.2, 1.0]	0.888
IV vs. I/II	-0.46	[-3.1, 2.2]	0.733
**Tumor size**	0.00	[-0.03, 0.03]	0.872

[1] Abbreviations: CI = Confidence Interval

Length of hospital stay (in days)

Table S2: Multivariable linear regression for length of hospital stay (in days), adjusting for surgical approach, age, sex, BMI, UICC-Stage, ASA Score and tumor size

**Characteristic**	**Mean difference**	**95% CI**[1]	**p-value**
**Surgical approach**			
Robotic vs Open	0.47	[-2.2, 3.2]	0.734
Laparoscopic vs. Open	0.33	[-2.2, 2.9]	0.798
**Sex**			
Males vs. Females	-0.63	[-2.8, 1.5]	0.561
**Age**	0.11	[0.01, 0.21]	0.038
**BMI**	-0.03	[-0.24, 0.19]	0.809
**UICC-Stage**			
Stage II vs. Stage 0/I	-2.8	[-6.1, 0.63]	0.109
Stage III vs. Stage 0/I	0.51	[-2.9, 3.9]	0.765
Stage IV vs. Stage 0/I	0.91	[-2.6, 4.5]	0.610
**ASA Score**			
III vs. I/II	1.2	[-1.3, 3.6]	0.342
IV vs. I/II	-1.2	[-7.1, 4.6]	0.681
**Tumor size**	0.04	[-0.03, 0.10]	0.251

[1] Abbreviations: CI = Confidence Interval

Postoperative CRP - day 1 (mg/dL)

Table S3: Multivariable linear regression for postoperative CRP - day 1 (mg/dL), adjusting for surgical approach, age, sex, BMI, UICC-Stage, ASA Score and tumor size

Characteristic	Mean difference	95% CI[1]	p-value
**Surgical approach**			
Robotic vs Open	-2.0	[-4.4, 0.47]	0.111
Laparoscopic vs. Open	-2.0	[-4.3, 0.29]	0.086
**Sex**			
Males vs. Females	-0.40	[-2.3, 1.5]	0.683
**Age**	-0.01	[-0.10, 0.09]	0.876
**BMI**	0.08	[-0.12, 0.27]	0.441
**UICC-Stage**			
Stage II vs. Stage 0/I	1.1	[-1.9, 4.2]	0.456
Stage III vs. Stage 0/I	2.1	[-0.94, 5.2]	0.171
Stage IV vs. Stage 0/I	2.6	[-0.56, 5.8]	0.105
**ASA Score**			
III vs. I/II	-0.58	[-2.8, 1.6]	0.605
IV vs. I/II	-1.1	[-6.4, 4.1]	0.672
**Tumor size**	0.00	[-0.05, 0.06]	0.909

[1] Abbreviations: CI = Confidence Interval

Duration of the operation (in minutes)

Table S4: Multivariable linear regression for duration of the operation (in minutes), adjusting for surgical approach, age, sex, BMI, UICC-Stage, ASA Score and tumor size

**Characteristic**	**Mean difference**	**95% CI[1]**	**p-value**
**Surgical approach**			
Robotic vs Open	50	[25, 75]	<0.001
Laparoscopic vs. Open	43	[19, 67]	<0.001
**Sex**			
Males vs. Females	-1.7	[-22, 18]	0.867
**Age**	-0.86	[-1.8, 0.11]	0.083
**BMI**	0.33	[-1.7, 2.4]	0.750
**UICC-Stage**			
Stage II vs. Stage 0/I	-20	[-52, 12]	0.211
Stage III vs. Stage 0/I	-9.7	[-41, 22]	0.546
Stage IV vs. Stage 0/I	-4.3	[-37, 29]	0.798
**ASA Score**			
III vs. I/II	-3.9	[-27, 19]	0.739
IV vs. I/II	11	[-44, 65]	0.703
**Tumor size**	0.44	[-0.16, 1.0]	0.149

[1] Abbreviations: CI = Confidence Interval

Lymph nodes yield

Table S5: Multivariable linear regression for lymph nodes yield, adjusting for surgical approach, age, sex, BMI, UICC-Stage, ASA Score and tumor size

**Characteristic**	**Mean difference**	**95% CI**[1]	**p-value**
**Surgical approach**			
Robotic vs Open	-1.9	[-8.2, 4.4]	0.555
Laparoscopic vs. Open	-3.8	[-9.7, 2.2]	0.214
**Sex**			
Males vs. Females	-0.29	[-5.3, 4.7]	0.906
**Age**	-0.25	[-0.50, -0.01]	0.041
**BMI**	0.12	[-0.39, 0.63]	0.634
**UICC-Stage**			
Stage II vs. Stage 0/I	4.7	[-3.1, 13]	0.236
Stage III vs. Stage 0/I	4.4	[-3.5, 12]	0.267
Stage IV vs. Stage 0/I	-5.6	[-14, 2.6]	0.178
**ASA Score**			
III vs. I/II	0.45	[-5.3, 6.2]	0.877
IV vs. I/II	-8.0	[-22, 5.6]	0.244
**Tumor size**	0.04	[-0.11, 0.19]	0.559

[1] Abbreviations: CI = Confidence Interval

CME quality

Table S6: Multivariable logistic regression for CME quality, adjusting for surgical approach, age, sex, BMI, UICC-Stage, ASA Score and tumor size

Characteristic	Odds Ratio	95% CI[1]	p-value
**Surgical approach**			
Robotic vs Open	0.58	[0.12, 2.56]	0.476
Laparoscopic vs. Open	1.16	[0.31, 4.49]	0.825
**Sex**			
Males vs. Females	0.37	[0.11, 1.16]	0.093
**Age**	0.98	[0.93, 1.05]	0.620
**BMI**	1.04	[0.92, 1.18]	0.503
**UICC-Stage**			
Stage II vs. Stage 0/I	4.89	[0.69, 49.1]	0.133
Stage III vs. Stage 0/I	3.23	[0.42, 33.7]	0.279
Stage IV vs. Stage 0/I	8.02	[1.07, 88.8]	0.058
**ASA Score**			
III vs. I/II	1.42	[0.32, 6.44]	0.643
IV vs. I/II	24.7	[0.99, 1,203]	0.066
**Tumor size**	1.00	[0.96, 1.03]	0.873

[1] Abbreviations: CI = Confidence Interval

Overall survival

Table S7: Multivariable Cox proportional hazards model for overall survival, adjusting for surgical approach, age, sex, BMI, UICC-Stage, ASA Score and tumor size

**Characteristic**	**Hazard Ratio**	**95% CI[1]**	**p-value**
**Surgical approach**			
Robotic vs Open	1.16	[0.39, 3.43]	0.786
Laparoscopic vs. Open	1.01	[0.31, 3.23]	0.993
**Sex**			
Males vs. Females	1.69	[0.64, 4.50]	0.293
**Age**	1.04	[0.98, 1.10]	0.219
**BMI**	1.03	[0.91, 1.17]	0.602
**UICC-Stage**			
Stage II vs. Stage 0/I	3.86	[0.40, 37.4]	0.243
Stage III vs. Stage 0/I	6.33	[0.65, 61.2]	0.111
Stage IV vs. Stage 0/I	17.3	[1.92, 156]	0.011
**ASA Score**			
III vs. I/II	1.80	[0.38, 8.46]	0.457
IV vs. I/II	3.39	[0.40, 28.9]	0.264
**Tumor size**	1.02	[0.99, 1.05]	0.170

[1] Abbreviations: CI = Confidence Interval

Disease-free survival

Table S8: Multivariable Cox proportional hazards model for disease-free survival, adjusting for surgical approach, age, sex, BMI, UICC-Stage, ASA Score and tumor size

Characteristic	Hazard Ratio	95% CI[1]	p-value
**Surgical approach**			
Robotic vs Open	0.89	[0.32, 2.53]	0.833
Laparoscopic vs. Open	0.88	[0.31, 2.52]	0.814
**Sex**			
Males vs. Females	1.73	[0.71, 4.20]	0.226
**Age**	1.00	[0.96, 1.05]	0.943
**BMI**	1.07	[0.97, 1.18]	0.153
**UICC-Stage**			
Stage II vs. Stage 0/I	2.16	[0.35, 13.3]	0.405
Stage III vs. Stage 0/I	3.96	[0.70, 22.5]	0.120
Stage IV vs. Stage 0/I	9.29	[1.77, 48.8]	0.008
**ASA Score**			
III vs. I/II	2.81	[0.75, 10.5]	0.124
IV vs. I/II	2.06	[0.30, 14.4]	0.464
**Tumor size**	1.02	[1.00, 1.05]	0.086

[1] Abbreviations: CI = Confidence Interval

Sensitivity analysis: Disease-free survival restricted to cases with UICC stages I–III

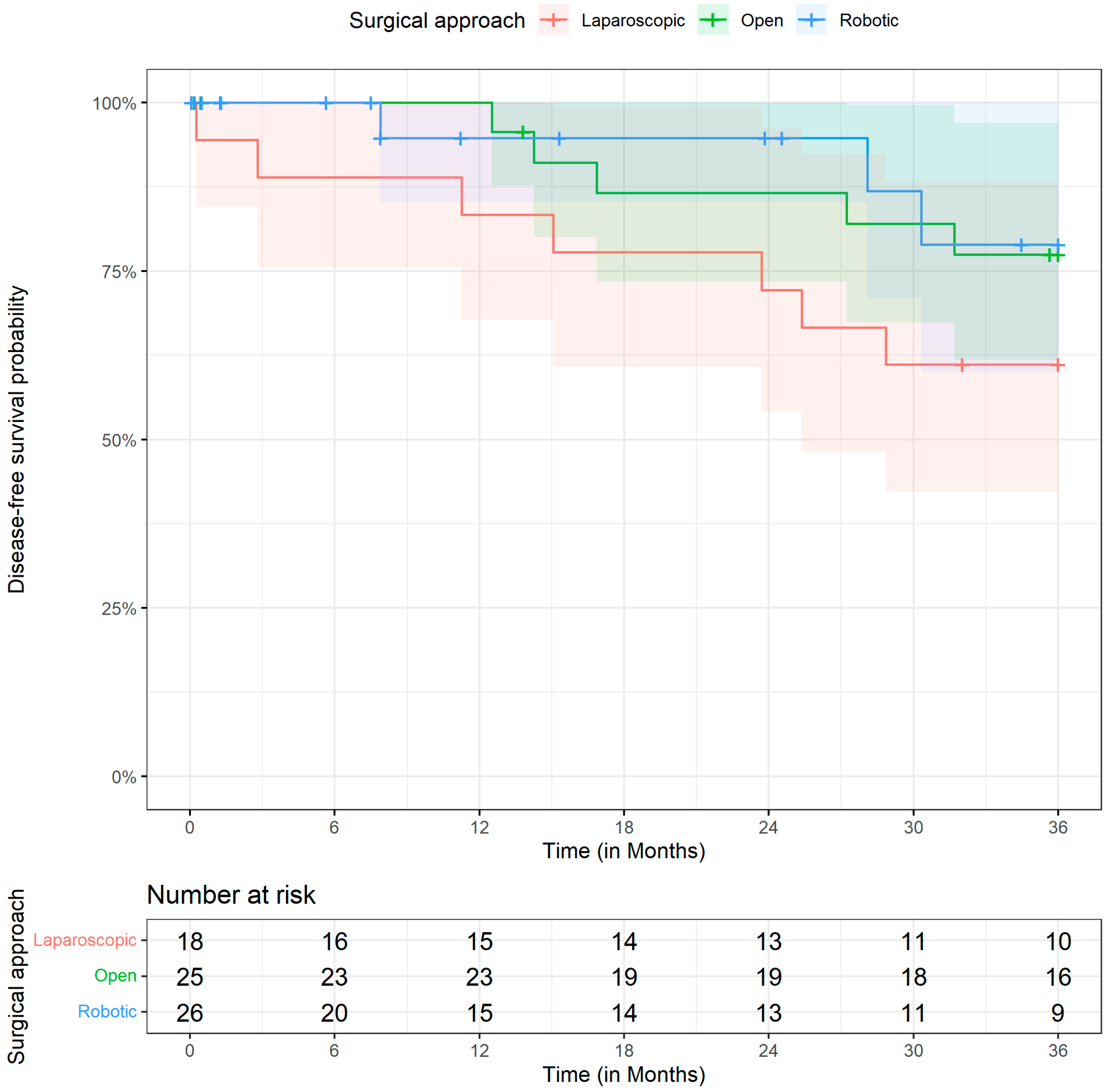

Figure S1: Disease-free survival in open, laparoscopic and robotic right colectomy groups restricted to cases with UICC stages I–III.

Table S9: Multivariable Cox proportional hazards model for disease-free survival, adjusting for surgical approach, age, sex, BMI, UICC-Stage, ASA Score and tumor size restricted to cases with UICC stages I–III

Characteristic	Hazard Ratio	95% CI[1]	p-value
**Surgical approach**			
Robotic vs Open	0.71	[0.14, 3.63]	0.676
Laparoscopic vs. Open	0.57	[0.11, 3.08]	0.515
**Sex**			
Males vs. Females	2.95	[0.73, 11.8]	0.127
**Age**	0.99	[0.93, 1.06]	0.807
**BMI**	1.05	[0.93, 1.18]	0.405
**UICC-Stage**			
Stage II vs. Stage 0/I	2.56	[0.41, 16.0]	0.316
Stage III vs. Stage 0/I	4.66	[0.75, 28.9]	0.099
**ASA Score**			
III vs. I/II	3.36	[0.79, 14.3]	0.100
IV vs. I/II	4.49	[0.27, 75.7]	0.298
**Tumor size**	1.03	[0.99, 1.06]	0.127

[1] Abbreviations: CI = Confidence Interval

Table S10: Kaplan–Meier estimates of overall survival including numbers at risk, events, and censoring over time

Time (Months)	Group	Number at risk	Events	Cumulative Events	Censored	Cumulative Censored	Survival probability (%)	95%-Confidence interval (%)
0	Open	30	0	0	0	0	100	[100; 100]
3		25	4	4	1	1	86	[75; 100]
6		25	0	4	0	1	86	[75; 100]
9		24	1	5	0	1	83	[70; 98]
12		24	0	5	0	1	83	[70; 98]
15		23	1	6	0	1	80	[66; 96]
18		23	0	6	0	1	80	[66; 96]
21		22	0	6	1	2	80	[66; 96]
24		21	1	7	0	2	76	[62; 93]
27		21	0	7	0	2	76	[62; 93]
30		21	0	7	0	2	76	[62; 93]
33		18	2	9	1	3	68	[53; 88]
36		16	2	11	16	19	61	[45; 82]
0	Robotic	30	0	0	0	0	100	[100; 100]
3		27	1	1	2	2	96	[90; 100]
6		27	0	1	0	2	96	[90; 100]
9		27	0	1	0	2	96	[90; 100]
12		27	0	1	0	2	96	[90; 100]
15		25	1	2	1	3	93	[84; 100]
18		22	3	5	0	3	82	[68; 98]
21		22	0	5	0	3	82	[68; 98]
24		21	0	5	1	4	82	[68; 98]
27		21	0	5	0	4	82	[68; 98]
30		20	1	6	0	4	78	[64; 95]
33		19	1	7	0	4	74	[59; 93]
36		17	1	8	18	22	70	[55; 90]
0	Laparoscopic	29	0	0	0	0	100	[100; 100]
3		24	0	0	5	5	100	[100; 100]
6		23	0	0	1	6	100	[100; 100]
9		20	0	0	3	9	100	[100; 100]
12		18	1	1	1	10	95	[86; 100]
15		18	0	1	0	10	95	[86; 100]
18		16	1	2	1	11	89	[77; 100]
21		15	1	3	0	11	84	[69; 100]
24		14	0	3	1	12	84	[69; 100]
27		13	0	3	1	13	84	[69; 100]
30		13	0	3	0	13	84	[69; 100]
33		13	0	3	0	13	84	[69; 100]
36		10	2	5	11	24	71	[52; 96]

Table S11: Kaplan–Meier estimates of disease-free survival including numbers at risk, events, and censoring over time

Time (Months)	Group	Number at risk	Events	Cumulative Events	Censored	Cumulative Censored	Survival probability (%)	95%-Confidence interval (%)
0	Open	30	0	0	0	0	100	[100; 100]
3		25	4	4	1	1	86	[75; 100]
6		24	1	5	0	1	83	[70; 98]
9		23	1	6	0	1	80	[66; 96]
12		22	1	7	0	1	76	[62; 93]
15		21	1	8	0	1	73	[58; 91]
18		20	1	9	0	1	69	[54; 88]
21		20	0	9	0	1	69	[54; 88]
24		19	1	10	0	1	66	[51; 85]
27		18	1	11	0	1	62	[47; 83]
30		16	2	13	0	1	55	[40; 77]
33		14	1	14	1	2	52	[36; 74]
36		14	0	14	14	16	52	[36; 74]
0	Robotic	30	0	0	0	0	100	[100; 100]
3		27	1	1	2	2	96	[90; 100]
6		27	0	1	0	2	96	[90; 100]
9		27	0	1	0	2	96	[90; 100]
12		27	0	1	0	2	96	[90; 100]
15		23	3	4	1	3	85	[73; 100]
18		20	3	7	0	3	74	[60; 93]
21		20	0	7	0	3	74	[60; 93]
24		20	0	7	0	3	74	[60; 93]
27		20	0	7	0	3	74	[60; 93]
30		19	1	8	0	3	71	[55; 90]
33		18	1	9	0	3	67	[51; 87]
36		17	0	9	18	21	67	[51; 87]
0	Laparoscopic	29	0	0	0	0	100	[100; 100]
3		23	1	1	5	5	96	[89; 100]
6		22	0	1	1	6	96	[89; 100]
9		18	1	2	3	9	92	[81; 100]
12		16	1	3	1	10	86	[73; 100]
15		16	0	3	0	10	86	[73; 100]
18		15	0	3	1	11	86	[73; 100]
21		15	0	3	0	11	86	[73; 100]
24		14	0	3	1	12	86	[73; 100]
27		13	0	3	1	13	86	[73; 100]
30		12	1	4	0	13	80	[64; 100]
33		11	1	5	0	13	73	[55; 97]
36		9	1	6	10	23	67	[47; 94]

In-hospital deaths

Table S12

Patient	Cause of death	Time to death (days)	Surgery-related death	Time to first bowel movement (days)
1	Aspiration	11	Yes	5
2	Unknown	8	Unknown	4

Patients with UICC stage IV disease

Table S13

	Open (N=12)	Robotic (N=5)	Laparoscopic (N=3)	Overall (N=20)
**UICC Stage**				
IV	12 (100%)	5 (100%)	3 (100%)	20 (100%)
**Tumor location**				
Ascending colon	7 (58.3%)	2 (40.0%)	2 (66.7%)	11 (55.0%)
Right colonic flexure	1 (8.3%)	1 (20.0%)	0 (0%)	2 (10.0%)
Cecum	4 (33.3%)	2 (40.0%)	1 (33.3%)	7 (35.0%)
**Tumor size (in mm)**				
Mean (SD)	43.5 (9.79)	58.4 (19.0)	50.3 (5.03)	48.3 (13.3)
Median [Q1, Q3]	43.5 [40.8, 49.0]	54.0 [42.0, 73.0]	51.0 [48.0, 53.0]	45.0 [41.0, 54.3]
**Surgical intent**				
Curative	5 (41.7%)	2 (40.0%)	2 (66.7%)	9 (45.0%)
Palliative	7 (58.3%)	3 (60.0%)	1 (33.3%)	11 (55.0%)
**Temporal classification**				
Synchronous	12 (100%)	5 (100%)	3 (100%)	20 (100%)
**Metastasectomy**				
None	7 (58.3%)	3 (60.0%)	0 (0%)	10 (50.0%)
Synchronous	2 (16.7%)	0 (0%)	0 (0%)	2 (10.0%)
Two-staged	3 (25.0%)	1 (20.0%)	2 (66.7%)	6 (30.0%)
Ablation	0 (0%)	1 (20.0%)	1 (33.3%)	2 (10.0%)
**Residual disease after colectomy**				
Yes	11 (91.7%)	5 (100%)	2 (66.7%)	18 (90.0%)
No	1 (8.3%)	0 (0%)	1 (33.3%)	2 (10.0%)
**No evidence of disease (NED) achieved**				
Yes	1 (8.3%)	0 (0%)	1 (33.3%)	2 (10.0%)
No	11 (91.7%)	5 (100%)	2 (66.7%)	18 (90.0%)

Distribution of procedures by year and surgeon

Table S14

Surgeon	Year	Open	Laparoscopic	Robotic
1	2021	4	0	0
2	2021	3	0	0
3	2019	0	1	0
4	2021	1	0	0
5	2021	1	0	0
6	2018	1	0	0
	2020	2	1	0
	2021	5	3	0
	2022	0	0	1
7	2020	1	0	0
	2021	1	0	0
8	2019	0	1	0
	2020	2	12	0
	2021	0	4	7
	2022	0	0	18
9	2019	1	0	0
	2020	1	0	0
	2022	0	1	3
10	2020	0	2	0
11	2018	1	0	0
	2021	1	0	0
12	2022	0	3	1
13	2021	1	0	0
14	2020	0	1	0
	2021	1	0	0
15	2020	3	0	0

Distribution of procedures by year

Table S15

Year	Open	Laparoscopic	Robotic
2018	2	0	0
2019	1	2	0
2020	9	16	0
2021	18	7	7
2022	0	4	23

Regression Diagnostics

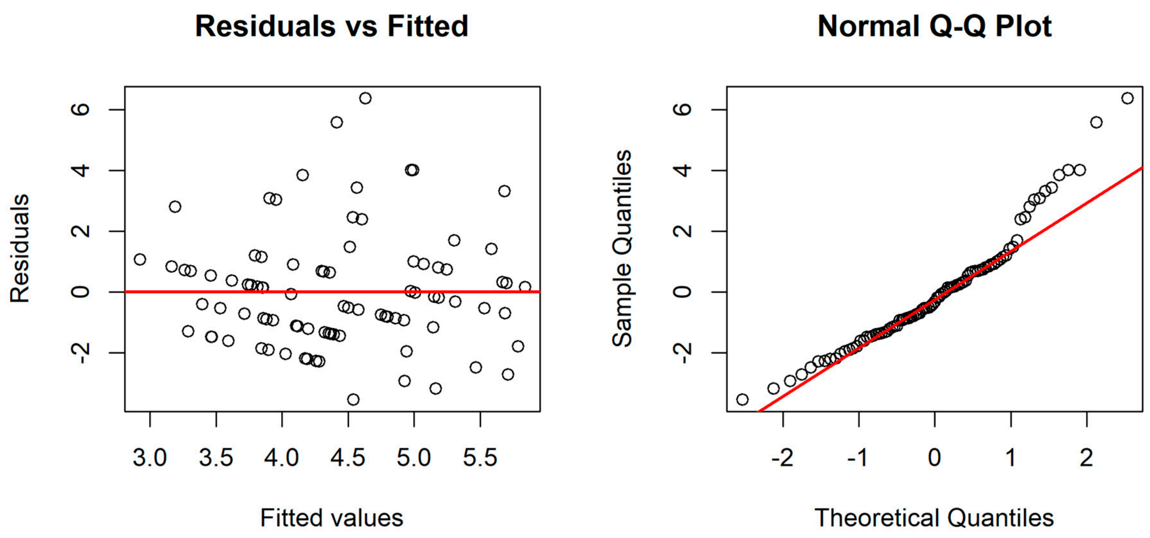

Figure S2: Regression Diagnostics – Postoperative bowel movement (in days)

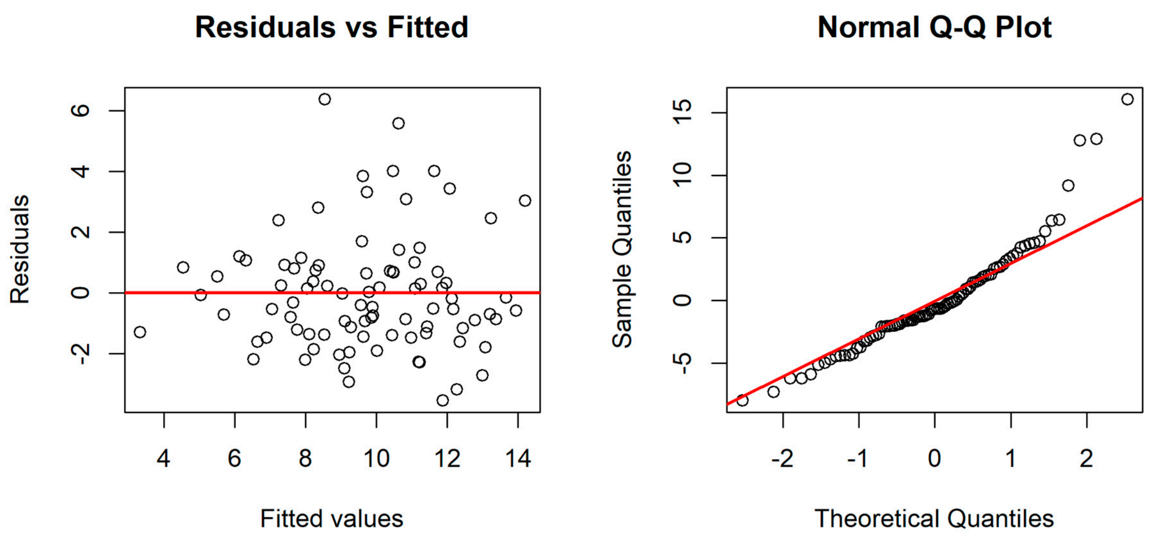

Figure S3: Regression Diagnostics – Length of stay (in days)
